# Patient‐Reported Outcome Measures Following Pulsed‐Field Ablation Versus Thermal Ablation of Atrial Fibrillation: The PROMs‐PFA Study

**DOI:** 10.1111/jce.70392

**Published:** 2026-06-09

**Authors:** Mark T. Mills, Peter Calvert, Keith Wilson, Zoltan Borbas, Richard Snowdon, Saagar N. Mahida, Reza Ashrafi, Johan Waktare, Simon Modi, Derick Todd, Nathan Denham, Vishal Luther, Dhiraj Gupta

**Affiliations:** ^1^ Department of Cardiology Liverpool Heart & Chest Hospital Liverpool Merseyside UK; ^2^ Liverpool Centre for Cardiovascular Science at University of Liverpool Liverpool John Moores University and Liverpool Heart & Chest Hospital Liverpool Merseyside UK; ^3^ Department of Cardiology Sheffield Teaching Hospitals NHS Foundation Trust Sheffield South Yorkshire UK; ^4^ Research Department, Liverpool Heart & Chest Hospital Liverpool Merseyside UK

**Keywords:** atrial fibrillation, catheter ablation, electroporation, patient‐reported outcome measures, pulmonary vein isolation, pulsed‐field ablation, symptoms

## Abstract

**Background:**

Pulsed‐field ablation (PFA) has transformed atrial fibrillation (AF) ablation, but how the early patient experience compares with thermal techniques remains unclear. This study aimed to assess patient‐reported outcome measures (PROMs) in the first 3 months after PFA or thermal AF ablation, using a novel smartphone‐based application.

**Methods:**

Patients undergoing first‐time pulmonary vein isolation prospectively completed the Cardiac Ablation Recovery Evaluation (CARE) symptom questionnaire at five timepoints: baseline, day 1, day 7, day 30, and day 90. Symptoms were rated on a 7‐point Likert scale (0 = not at all; 6 = extremely). Patients also completed the Atrial Fibrillation Effect on QualiTy‐of‐life (AFEQT) questionnaire at baseline and day 90.

**Results:**

Two hundred and two patients were included (pentaspline PFA: 66; radiofrequency: 64; cryoballoon: 72), with similar baseline demographics between groups, except for a lower prevalence of paroxysmal AF in the PFA group. Engagement with the smartphone‐based platform was high, with a 94.7% completion rate. Median AFEQT scores improved from 64 (47–82) at baseline to 86 (72–95) at day 90 (*p* < 0.001), with no significant differences between modalities (*p* = 0.841). However, CARE detected differences in early symptom experience: chest pain was lowest with PFA at day 1 (median: PFA, 1 [0–2]; radiofrequency, 2 [1–2]; cryoballoon, 2 [1–2]; *p* < 0.001), and remained lower than cryoballoon at day 7 (*p* = 0.019), with differences resolving by day 30. Moderate‐to‐severe chest pain (score ≥ 3) occurred in only 12.3% of PFA patients at day 1, compared with 21.2% cryoballoon and 26.2% radiofrequency patients (*p* < 0.001). Palpitations at day 1 were less frequent with PFA than radiofrequency (*p* = 0.040). Groin discomfort was lower with radiofrequency than PFA or cryoballoon at day 1 (*p* = 0.006) and day 7 (*p* < 0.001), likely reflecting a smaller sheath size.

**Conclusions:**

PFA was associated with a modest and transient reduction in early post‐procedural chest pain compared with thermal ablation. Smartphone application‐based PROMs collection using the CARE questionnaire was feasible, enabling structured assessment of symptom burden across multiple recovery timepoints.

## Graphical abstract

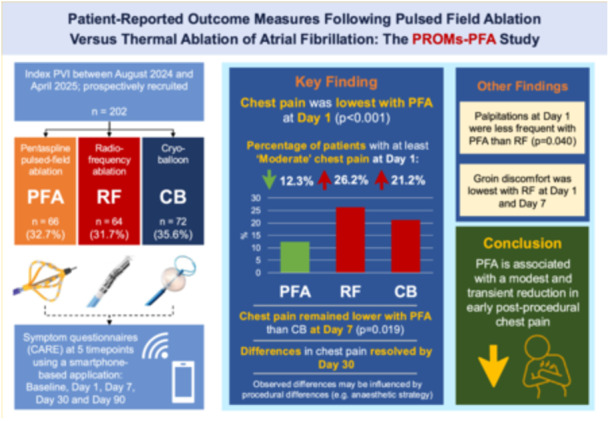

## 1 Introduction

Catheter ablation is an established treatment for atrial fibrillation (AF), with increasing global uptake. Traditionally, thermal energy sources such as radiofrequency (RF) and cryoballoon (CB) have been used to achieve pulmonary vein isolation (PVI), the cornerstone of AF ablation [1, 2]. More recently, pulsed‐field ablation (PFA), a non‐thermal modality that selectively targets myocardial cells through electroporation, has emerged as a promising alternative [3, 4, 5, 6]. As adoption of PFA accelerates, there is growing interest in its impact on the patient experience.

Early post‐procedural symptoms following AF ablation are common and may include chest pain, palpitations, dyspnoea, headache, gastrointestinal disturbance and mood disturbance [7, 8]. These symptoms are thought to arise from a multitude of factors, including thermal injury to cardiac or extracardiac structures, transient (or persistent) arrhythmia recurrence, and autonomic dysregulation [8, 9, 10]. Although typically self‐limiting, these early symptoms can impact recovery, lead to repeat medical contact and affect patient satisfaction with the procedure [11]. Anecdotal observations and small studies suggest that patients undergoing PFA may experience fewer or milder early post‐ablation symptoms—particularly chest pain—potentially reflecting its cardioselectivity and reduced collateral damage [12, 13].

Despite the importance of patient‐reported outcome measures (PROMs), data on symptom burden and recovery trajectories following ablation are sparse, particularly in the context of newer technologies like PFA. Existing validated AF symptom and quality‐of‐life tools were developed to assess medium‐ to long‐term outcomes and may not adequately capture early post‐procedural recovery [14, 15]. There is therefore a need for instruments tailored to the immediate recovery phase to enable meaningful comparisons across ablation modalities. The PROMs Following Pulsed‐Field Ablation Versus Thermal Ablation of Atrial Fibrillation (PROMs‐PFA) study aimed to systematically compare patient‐reported symptoms in the first 3 months following PFA, RF, and CB ablation for AF using a novel symptom questionnaire delivered via a smartphone‐based application.

## 2 Methods

### 2.1 Study Design

Patients scheduled for index PVI using one of three ablation modalities (PFA, RF or CB) at Liverpool Heart and Chest Hospital (LHCH) between 14th August 2024 and 29th April 2025 were prospectively invited to participate. Invitations were sent via letter and text message between 1 month and 1 week prior to the planned procedure. These communications outlined the study aims and timeline, with patients asked to download a smartphone application and report symptoms at five specific timepoints: baseline (between 30 days and 1 day prior to ablation), and on Day 1, Day 7, Day 30, and Day 90 following the ablation procedure (Day 0).

Interested patients were instructed to download a bespoke smartphone application (Treatpath, Buddy Healthcare, see Supporting Material—Figure S1), which was used for data collection, where they could complete the baseline symptom questionnaire. Reminder notifications were sent through the app and through text messages. Follow‐up phone calls were used where necessary to encourage completion of the baseline questionnaire prior to ablation and to prompt submission of follow‐up questionnaires if patients had not responded to automated reminders. Patients who did not complete the baseline questionnaire prior to their procedure were not contacted further.

All participants provided written consent for their clinical procedure. The project was approved by the LHCH Research & Innovation Committee. As a prospective, observational study, PROMs‐PFA is reported according to the Strengthening the Reporting of Observational Studies in Epidemiology (STROBE) guidelines. The data that support the findings of this study are available from the corresponding author upon reasonable request.

### 2.2 Assessment of Patient‐Reported Outcome Measures and Development of Novel Questionnaire

Patients were asked to complete the validated Atrial Fibrillation Effect on QualiTy‐of‐life (AFEQT) questionnaire [14] at two timepoints (baseline and Day 90).

In addition to the AFEQT questionnaire, patients completed a novel symptom questionnaire at each of the five study timepoints (baseline, Days 1, 7, 30 and 90). This questionnaire, termed the Cardiac Ablation Recovery Evaluation (CARE) questionnaire, was developed by the LHCH Electrophysiology Research Group to comprehensively assess the range of symptoms patients may experience following AF ablation. As no existing instruments specifically assess early post‐ablation symptom burden, an initial list of symptoms was generated based on clinical experience and commonly observed post‐procedural issues following AF ablation. The draft questionnaire was subsequently reviewed by the study investigators and the institutional Research & Innovation department, and feedback was obtained from 10 patients who had recently undergone AF ablation to assess relevance and clarity. The questionnaire was then refined further, including modification of symptom items and adjustments to layout and terminology, based on patient feedback and input from an experienced Patient Research Representative (KW).

The selected symptoms in the CARE questionnaire were chosen to capture those potentially related to procedural adverse events (e.g. myocardial/pericardial inflammation, vagus nerve injury, transseptal access), arrhythmia recurrence, autonomic disturbance, or the general recovery process. These included: cardiac (chest pain or discomfort, palpitations, dizziness or light‐headedness), respiratory (dyspnoea), neurological/cognitive (headache, visual disturbance, brain fog), gastrointestinal (nausea, vomiting, bloating, early satiety, change in bowel habit, diarrhoea, constipation, and difficulty swallowing), psychological (low mood, anxiety), and miscellaneous (difficulty voiding the bladder and groin pain; the latter assessed from D1 onwards only).

Each symptom was rated on a 7‐point Likert scale (0–6), using consistent language with the AFEQT questionnaire (0 = Not at all, 1 = Hardly, 2 = A little, 3 = Moderately, 4 = Quite a bit, 5 = Very, 6 = Extremely). The CARE questionnaire is provided in Supporting Material—Table S1.

To avoid overlap in symptom reporting, patients were instructed to report symptoms over specific preceding intervals: the baseline questionnaire captured symptoms over the prior 4 weeks; Day 1 captured symptoms experienced since the ablation; Day 7 covered the preceding 6 days; Day 30 the preceding 3 weeks; and Day 90 the preceding 4 weeks.

The AFEQT and CARE questionnaires were subsequently integrated into the TreatPath application. This digital platform facilitated real‐time data collection and patient engagement (e.g. through ‘push notifications’), while ensuring consistency in symptom assessment across all five timepoints.

### 2.3 Ablation Procedures

The technical approaches to RF, CB, and PFA at our centre have been detailed previously [16, 17, 18, 19]. In brief, PFA was undertaken with the pentaspline FARAPULSE system (Boston Scientific), following the manufacturer's instructions and the procedural framework described in the 5S study [20]. Each pulmonary vein (PV) received a minimum of eight applications at 2.0 kW—four in the basket configuration and four in the flower configuration. Following this standard lesion set, additional PV applications were permitted at operator discretion (e.g. if acute PVI was not achieved). RF ablation was predominantly carried out using the ThermoCool SmartTouch catheter in conjunction with the CARTO electroanatomic mapping system (J&J MedTech), at 50 W power. CB procedures were performed using the Arctic Front Advance 28 mm balloon catheter (Medtronic). Both PFA and CB procedures were conducted under fluoroscopic guidance without the use of 3D electroanatomic mapping.

In the majority of patients, ablation was limited to isolation of the PVs, regardless of whether AF was paroxysmal or persistent. At the operator's discretion, adjunctive posterior wall (PW) ablation was performed in a subset of patients with persistent or long‐standing persistent AF. In the PFA cohort, this was achieved using the flower configuration of the FARAWAVE catheter, with the number of lesions tailored to the size of the PW. Cavotricuspid isthmus (CTI) ablation was performed in cases where sustained atrial flutter was induced during the procedure or previously documented. This necessitated the use of an additional RF catheter in patients undergoing CB or PFA.

General anaesthesia (GA) with intubation and ventilation was employed for all PFA cases due to the potential for painful musculoskeletal responses. In contrast, GA use for RF and CB ablation was determined by operator judgement or patient preference [16, 18, 21]. For non‐GA cases, mild conscious sedation was administered without anaesthetic support, using operator‐determined doses of midazolam and fentanyl.

Procedures were performed by 10 consultant electrophysiologists with experience of PFA using the pentaspline catheter since 2022 [5, 18, 21, 22, 23]. As one of the few centres offering all three ablation modalities in broadly equal numbers, our single‐centre setting provided a valuable opportunity to compare real‐world procedural workflows and patient experiences across approaches. As this was an observational study, ablation modality was not randomised. The choice of technique reflected routine clinical practice and was influenced by operator preference, patient preference (particularly regarding the use of GA vs. conscious sedation) and, in certain circumstances, clinical phenotype (e.g. anticipated need for adjunctive substrate modification).

### 2.4 Statistical Analysis

Patients were included in the analysis if they completed at least two of the five questionnaires, one of which had to be the baseline assessment. Incomplete questionnaires at specific time points were excluded from the relevant analysis, and the resulting sample sizes are reported.

Data distribution was assessed visually (via histograms) and statistically using the Shapiro–Wilk test. Continuous variables are reported as mean ± standard deviation (SD) for normally distributed data, or as median and interquartile range [IQR: 25th–75th percentile] for non‐normally distributed data. Categorical variables are summarised as frequencies and percentages, and compared using the Chi‐square test. Paired continuous variables were compared using a paired samples *t*‐test or a Wilcoxon signed‐rank test, as appropriate. Continuous variables between three independent groups were compared using one‐way ANOVA or the Kruskal–Wallis test, as appropriate; where significant differences were identified, post hoc pairwise comparisons were conducted with Bonferroni correction to adjust for multiple testing.

Given the imbalance in anaesthetic strategy between ablation modalities, multi‐variable adjustment was considered inappropriate because of substantial collinearity between anaesthesia type and treatment group. Instead, exploratory stratified analyses were performed for chest pain according to GA versus conscious sedation, including within the RF sub‐group where both anaesthetic approaches were represented.

All analyses were performed using IBM SPSS Statistics (Version 29.0.1.0, IBM Corp). A two‐tailed *p*‐value < 0.05 was considered statistically significant.

## 3 Results

### 3.1 Baseline Characteristics

**Figure 1:**
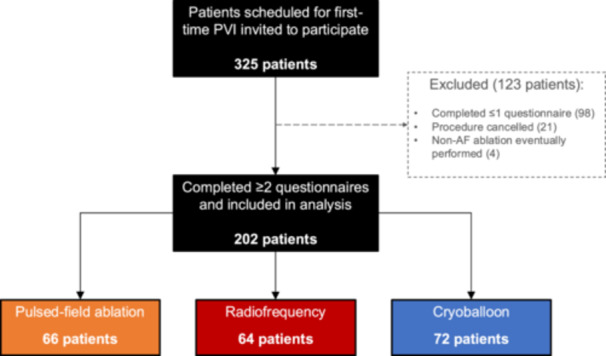
Study flowchart. AF, atrial fibrillation; PVI, pulmonary vein isolation.

A total of 202 patients were included in the analysis (Figure 1). Of these, 66 (32.7%) underwent PFA, 64 (31.7%) RF ablation, and 72 (35.6%) CB ablation. Baseline demographics and clinical characteristics are summarised in Table 1. Across the cohort, 32.7% were female, the median age was 63 years (56–70), median body mass index was 28.5 kg/m^2^ (25.8–31.7), and the median CHA_2_DS_2_‐VASc score was 2 [1–2]. The three groups were comparable for all recorded characteristics—including age, sex, comorbidities, CHA_2_DS_2_‐VASc score and pre‐ablation anti‐arrhythmic drug use—with the exception of paroxysmal AF, which was less common in the PFA group (PFA 39.4%, RF 62.5%, CB 63.9%; *p* = 0.006) and moderate or severe left atrial dilatation which was more common in the PFA group (PFA 48.5%, RF 29.7%, CB 26.4%; *p* = 0.015).

**Table 1:** Baseline demographics and clinical characteristics across ablation modalities.

Characteristic	PFA (*n* = 66)	RF (*n* = 64)	CB (*n* = 72)	*p* value
Age, years	63 [54–69]	63 [55–72]	63 [57–70]	0.676
Female sex, *n* (%)	22 (33.3%)	22 (34.3%)	22 (30.6%)	0.885
BMI, kg/m^2^	30 [26–32]	28 [25–31]	29 [26–31]	0.342
AF type				**0.006**
Paroxysmal, *n* (%)	**26 (39.4%)**	40 (62.5%)	46 (63.9%)	
Persistent, *n* (%)	**40 (60.6%)**	24 (37.5%)	26 (36.1%)	
Heart failure, *n* (%)	14 (21.2%)	11 (17.2%)	20 (27.8%)	0.323
Hypertension, *n* (%)	30 (45.5%)	29 (45.3%)	37 (51.4%)	0.715
Stroke/TIA, *n* (%)	8 (12.1%)	3 (4.7%)	8 (11.1%)	0.288
Ischaemic heart disease, *n* (%)	4 (6.1%)	3 (4.7%)	7 (9.7%)	0.485
Aortic or peripheral vascular disease, *n* (%)	2 (3.0%)	1 (1.6%)	0 (0%)	0.339
Diabetes mellitus, *n* (%)	6 (9.1%)	7 (10.9%)	8 (11.1%)	0.914
CHA_2_DS_2_‐VASc score	2 [1–2]	2 [1–3]	2 [1–2]	0.643
eGFR, mL/min/1.73 m^2^	76 [65–90]	78 [63–88]	85 [70–90]	0.140
Moderate or severe LA dilatation, *n* (%)	**32 (48.5%)**	19 (29.7%)	19 (26.4%)	**0.015**
Moderate or severe LV dysfunction, *n* (%)	8 (12.1%)	4 (6.3%)	9 (12.5%)	0.420
AAD use before ablation, *n* (%)	20 (30.3%)	23 (35.9%)	28 (38.9%)	0.566

Abbreviations: AAD, anti‐arrhythmic drug; AF, atrial fibrillation; BMI, body mass index; CB, cryoballoon; eGFR, estimated glomerular filtration rate; LA, left atrial; LV, left ventricular; PFA, pulsed‐field ablation; RF, radiofrequency; TIA, transient ischaemic attack. *NOTE:* Bold values are statistically significant differences.

### 3.2 Procedural Characteristics

Procedural details are summarised in Table 2. Acute procedural success, defined as isolation of all PVs, was achieved in 100% of PFA and RF cases, but only 94.4% of CB cases (*p* = 0.025). All PFA procedures were performed under GA, compared with 68.8% of RF procedures and just one CB case (1.4%) (*p* < 0.001). Transoesophageal echocardiography (TOE) was utilised in 62.1% of PFA cases and 65.6% of RF cases, but in none of the CB cases (*p* < 0.001). Non‐PV ablation was performed in 25.8% of PFA cases, 21.9% of RF cases, and 5.6% of CB cases (*p* = 0.004). This was primarily driven by concomitant CTI ablation in RF procedures (21.9%) and PW ablation in PFA cases (21.2%). Skin‐to‐skin procedure time was significantly shorter with PFA than with RF or CB ablation, whereas fluoroscopy time was lower with RF compared with both single‐shot modalities.

**Table 2:** Procedural characteristics across ablation modalities.

Characteristic	PFA (*n* = 66)	RF (*n* = 64)	CB (*n* = 72)	*p* value
AF at procedure start, *n* (%)	29 (43.9%)	21 (32.8%)	**10 (13.9%)**	**< 0.001**
General anaesthesia, *n* (%)	66 (100%)	44 (68.8%)	**1 (1.4%)**	**< 0.001**
Transoesophageal echocardiography guidance, *n* (%)	41 (62.1%)	42 (65.6%)	**0 (0%)**	**< 0.001**
Ultrasound‐guided access, *n* (%)	66 (100%)	62 (96.9%)	68 (94.4%)	0.158
DCCV during procedure, *n* (%)	**29 (43.9%)**	18 (28.1%)	12 (16.7%)	**0.002**
Non‐PV procedure performed, *n* (%)^a^	17 (25.8%)	14 (21.9%)	**4 (5.6%)**	**0.004**
CTI	4 (6.1%)	**14 (21.9%)**	4 (5.6%)	**0.003**
PW ablation	**14 (21.2%)**	1 (1.6%)	0 (0%)	< **0.001**
LA linear ablation	0 (0%)	1 (1.6%)	0 (0%)	0.338
Acute procedural success, *n* (%)	66 (100%)	64 (100%)	**68 (94.4%)**	**0.025**
Major complication, *n* (%)	0 (0%)	1 (1.6%)	1 (1.4%)	0.609

a Certain patients received non‐PV ablation in more than one area (e.g. CTI and PW ablation)

Abbreviations: AF, atrial fibrillation; CB, cryoballoon; CTI, cavotricuspid isthmus; DCCV, direct current cardioversion; LA, left atrial; PFA, pulsed‐field ablation; PV, pulmonary vein; PW, posterior wall; RF, radiofrequency. *NOTE:* Bold values are statistically significant differences.

Two major complications occurred (1.0%): one case of pericardial effusion requiring percutaneous drainage in the RF group, and one case of persistent phrenic nerve palsy in a patient treated with CB.

### 3.3 Patient‐Reported Outcomes Measures: General Trends

Engagement with the smartphone‐based platform was high, with a 94.7% (956/1010) completion rate across the five questionnaires. Of the 202 patients, all completed the baseline questionnaire, 192 (95.0%) Day 1, 188 (93.1%) Day 7, 188 (93.1%) Day 30, and 186 (92.1%) Day 90. Overall, 167 (82.7%) patients completed all 5 questionnaires, 21 (10.4%) completed 4 questionnaires, 9 (4.5%) completed 3 questionnaires, and 5 (2.5%) completed 2 questionnaires.

Between baseline and Day 90, overall AFEQT scores improved from 64 [47–82] to 86 [72–95] (*p* < 0.001), with improvements observed across all subscales (see Supporting Material—Table S2).

Across the whole cohort, the two most common symptoms at Day 1 were chest pain/discomfort (median score 2 [0–2]) and groin pain (2 [1–3]). These symptoms improved over time, with scores falling to 1 [0–2] for both by Day 7. By Day 30, groin discomfort had further improved to 0 [0–1], while chest pain remained at 1 [0–2]; by Day 90, median scores were 0 [0–1] for chest pain and 0 [0–0] for groin discomfort.

The trajectory of chest pain scores across the cohort is summarised in Supporting Material—Figure S2. At baseline, 43.1% of patients reported no chest pain, while 15.8% reported “Hardly any,” and 19.3% “A little.” The proportion of patients reporting at least “Moderate” chest pain (score ≥ 3) was 21.8% at baseline, 19.8% on Day 1, gradually declining to 16.9% on Day 7, 16.5% on Day 30, and 10.3% by Day 90. The proportion reporting “No” or “Hardly any” chest pain increased progressively from 49.0% on Day 1% to 75.8% by Day 90. “Very” or “Extremely” severe chest pain remained uncommon at all timepoints, peaking at 4.8% on Day 30.

Palpitations (“awareness of a fast or abnormal heart rate”) improved following ablation, with a median score of 3 [1–4] at baseline; 0 [0–2] on Day 1; 1 [0–2] on Day 7; 1 [0–2] on Day 30; and 1 [0–2] on Day 90. Dyspnoea scores followed a similar pattern, with scores of 2 [0–3] at baseline; 0 [0–1] on Day 1; 1 [0–2] on Day 7; 1 [0–2] on Day 30; and 1 [0–2] on Day 90.

Aside from chest pain, groin discomfort, palpitations and dyspnoea, all other recorded symptoms – including gastrointestinal and neurological symptoms – were infrequently reported, with median scores ≤ 1 at all timepoints.

### 3.4 Symptoms by Ablation Modality

No significant differences were observed in overall AFEQT scores or in individual subscale domains between the three ablation modalities at baseline or Day 90 (Table 3). Patient‐reported symptom differences across modalities are summarised in Table 4.

**Table 3:** Atrial fibrillation effect on quality‐of‐life (AFEQT) score by ablation modality.

AFEQT score	PFA	RF	CB	*p* value
Baseline AFEQT				
Symptom score	71 [55–83]	75 [57–89]	75 [54–92]	0.713
Activity score	66 [38–90]	71 [41–92]	61 [40–92]	0.931
Treatment concern	63 [39–75]	67 [44–78]	65 [47–78]	0.517
Treatment satisfaction	50 [25–67]	42 [23–67]	54 [25–83]	0.168
Overall score	63 [46–82]	66 [46–82]	63 [47–82]	0.752
Day 90 AFEQT				
Symptom score	92 [79–100]	92 [79–100]	92 [75–100]	0.920
Activity score	85 [69–97]	90 [71–100]	88 [64–98]	0.843
Treatment concern	83 [70–94]	86 [67–97]	88 [72–98]	0.637
Treatment satisfaction	83 [75–100]	83 [67–100]	83 [58–100]	0.768
Overall score	84 [74–94]	88 [74–97]	86 [70–96]	0.841

Abbreviations: CB, cryoballoon; PFA, pulsed‐field ablation; RF, radiofrequency.

**Table 4:** Differences in post‐procedural symptoms across ablation modalities.

Symptom	PFA	RF	CB	*p* value
Chest pain or discomfort				
Baseline	2 [0–3]	1 [0–2]	1 [0–2]	0.194
Day‐1	**1 [0–2]**	2 [1–2]	2 [1–2]	**< 0.001**
Day‐7	1 [0–2]	1 [0–2]	**2 [0–2]**	**0.024**
Day‐30	0 [0–2]	2 [0–2]	1 [0–2]	0.165
Day‐90	0 [0–1]	0 [0–1]	0 [0–1.5]	0.665
Palpitations				
Baseline	3 [1–4]	3 [1–4]	3 [1–4]	0.983
Day‐1	**0 [0–1.25]**	1 [0–2]	1 [0–2]	**0.046**
Day‐7	1 [0–2]	1 [0–2]	2 [0–3]	0.070
Day‐30	1 [0–2]	2 [0–3]	2 [0–3]	0.540
Day‐90	0.5 [0–2]	1 [0–2]	1 [0–3]	0.354
Groin discomfort				
Baseline	N/A	N/A	N/A	
Day‐1	2 [1.25–2.25]	**1 [0–2]**	2 [1–3]	**0.006**
Day‐7	1 [1–2]	0 [0–2]	**2 [1–3]**	**< 0.001**
Day‐30	1 [0–1]	0 [0–1]	0 [0–1]	0.418
Day‐90	0 [0–0]	0 [0–0]	0 [0–0]	0.749
Difficulty swallowing				
Baseline	0 [0–0.25]	0 [0–0]	0 [0–0]	0.748
Day‐1	2 [0–2]	1 [0–2]	**0 [0–1]**	**< 0.001**
Day‐7	0 [0–1]	0 [0–1]	**0 [0–0]**	**0.012**
Day‐30	0 [0–0.25]	0 [0–1]	0 [0–0]	0.326
Day‐90	0 [0–0]	0 [0–0]	0 [0–0]	0.233
Early satiety				
Baseline	1 [0–2]	0 [0–1]	0 [0–2]	0.085
Day‐1	1 [0–2]	1 [0–2]	**0 [0–1]**	**0.045**
Day‐7	0 [0–2]	0 [0–2]	0 [0–1]	0.727
Day‐30	0 [0–2]	0 [0–2]	0 [0–2]	0.580
Day‐90	0 [0–1]	0 [0–1]	0 [0–1]	0.865
Difficulty voiding bladder				
Baseline	**0 [0–2]**	0 [0–0]	0 [0–0]	**0.043**
Day‐1	0 [0–1]	0 [0–1]	**0 [0–0]**	**0.009**
Day‐7	**0 [0–1]**	0 [0–0]	0 [0–0]	**0.040**
Day‐30	0 [0–1]	0 [0–0]	0 [0–0]	0.434
Day‐90	0 [0–1]	0 [0–1]	0 [0–0]	0.430

*Note:* p‐values are corrected for multiple hypothesis testing via the Bonferroni method. Bold values are statistically significant differences.

Abbreviations: CB, cryoballoon; PFA, pulsed‐field ablation; RF, radiofrequency.

**Figure 2:**
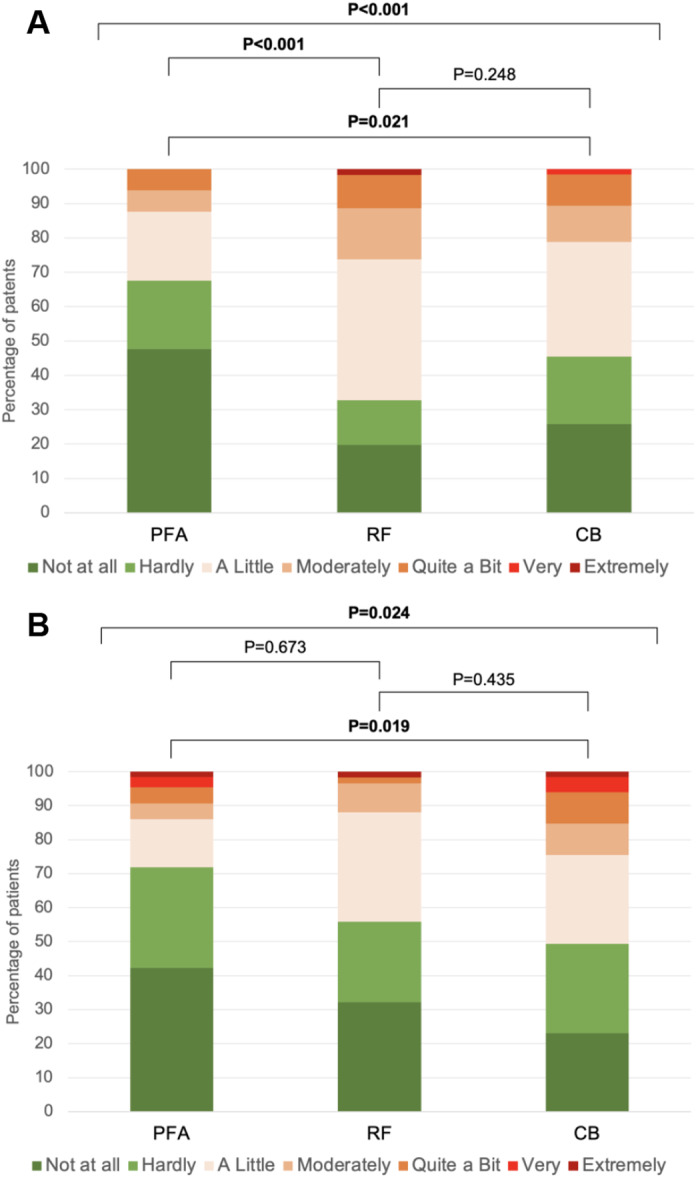
Differences in chest pain at Day 1 (A) and Day 7 (B) by ablation modality. CB, cryoballoon; PFA, pulsed‐field ablation; RF, radiofrequency.

Chest pain or discomfort was significantly lower in the PFA group at Day 1 (median [IQR]: PFA 1 [0–2], RF 2 [1–2], CB 2 [1–2]; *p* < 0.001) and Day 7 (PFA 1 [0–2], RF 1 [0–2], CB 2 [0–2]; *p* = 0.024). Differences in chest pain had resolved by Day 30 (Table 4). A breakdown of chest pain scores at Days 1 and 7 is shown in Figure 2. At Day 1, 67.9% (44/65) of patients in the PFA group reported “No” or “Hardly any” chest pain, compared with 32.9% (20/61) in the RF group and 45.5% (30/66) in the CB group (*p* < 0.001). By Day 7, the proportions were 71.9% (46/64) for PFA, 55.9% (33/59) for RF, and 49.2% (32/65) for CB (*p* = 0.019 for PFA vs. CB). Sensitivity analysis excluding patients who underwent concomitant CTI ablation confirmed that these differences remained significant at Day 1 (*p* < 0.001 across modalities) and Day 7 (*p* = 0.011). Similarly, sensitivity analysis excluding patients who underwent PW ablation demonstrated consistent findings, with significantly lower chest pain scores in the PFA group at both Day 1 (*p* < 0.001 across modalities) and Day 7 (*p* = 0.018).

Notably, 19.8% of patients (38/192) had a Day 1 chest pain score ≥ 3 (i.e. at least “Moderate”); all had undergone technically uncomplicated procedures, except one patient in the CB group who sustained a phrenic nerve palsy, and no consistent association between higher symptom scores and procedural complications was observed. The incidence of at least “Moderate” chest pain was lower in the PFA cohort (12.3%; 8/65) compared with the CB (21.2%; 14/66) and RF (26.2%; 16/61) cohorts (*p* < 0.001). The highest reported chest pain score at Day 1 was 4 (“Quite a Bit”) in the PFA group, compared with 5 (“Very”) in the CB group and 6 (“Extremely”) in the RF group (Figure 2).

**Figure 3:**
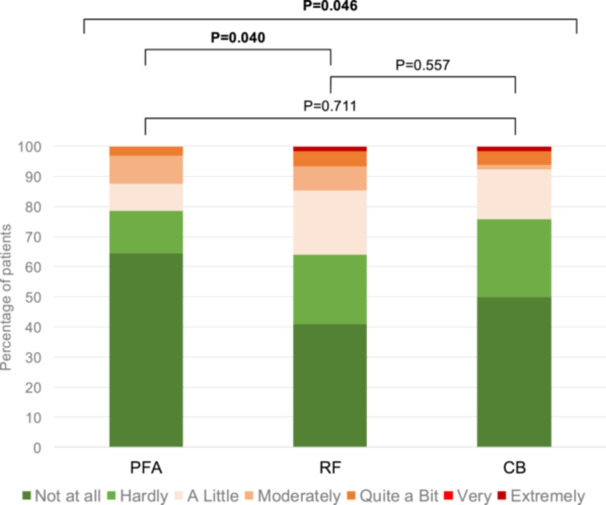
Differences in palpitations at Day 1 by ablation modality. CB, cryoballoon; PFA, pulsed‐field ablation; RF, radiofrequency.

Palpitations were also less frequent in the PFA group at Day 1 (*p* = 0.046 across groups; *p* = 0.040 for PFA vs. RF), although this difference did not persist to Day 7 (Table 4 and Figure 3). Groin discomfort was lowest in the RF group at both Day 1 (RF 1 [0–2] vs PFA 2 [1.25–2.25] and CB 2 [1–3]; *p* = 0.006) and Day 7 (*p* < 0.001), likely reflecting smaller sheath size (Table 4).

Difficulty swallowing was more frequently reported in the RF and PFA groups at Day 1 (PFA 2 [0–2], RF 1 [0–2], CB 0 [0–1]; *p* < 0.001), and persisted at Day 7 (*p* = 0.012). This likely reflects greater use of TOE rather than a modality‐specific adverse event, as patients who underwent TOE (*n* = 83) reported more difficulty swallowing at Day 1 (median score 2 [0–2]) compared to those without TOE (*n* = 119; 0 [0–1]) (*p* < 0.001). Early satiety and difficulty voiding the bladder were infrequently reported but showed transient differences at Day 1 (*p* = 0.045 and *p* = 0.009, respectively), with no persistent differences beyond the first week (Table 4).

No significant between‐group differences were observed at any timepoint for light‐headedness, dyspnoea, heartburn, bloating, nausea, vomiting, constipation, diarrhoea, headache, brain fog, visual disturbance, low mood or anxiety (see Supporting Material —Table S3).

### 3.5 Symptoms by Anaesthesia Use

Because GA use differed substantially between modalities, an exploratory analysis was performed to assess whether early chest pain varied according to anaesthetic strategy (Table 5). In the overall cohort, Day 1 chest pain scores were lower in patients treated under GA than in those treated with conscious sedation (median [IQR]: 1 [0–2] vs. 2 [1–2]; *p* = 0.021), although the proportion of patients reporting at least moderate chest pain (score ≥ 3) did not differ significantly (15.9% vs. 24.7%; *p* = 0.180) (Table 5, Panel A). Within the RF sub‐group, where both anaesthetic strategies were used, there were no statistically significant differences in median chest pain scores between patients treated under GA and those treated with conscious sedation (median [IQR]: 2 [1–2] vs. 2 [2–3]; *p* = 0.084), nor in the proportion reporting moderate or greater chest pain (19.0% vs. 36.8%; *p* = 0.136) (Table 5, Panel B).

**Table 5:** Chest pain at Day 1 stratified by anaesthetic strategy.

**Panel A—Whole cohort**			
Variable	General anaesthesia(N = 107)	Mild conscious sedation(N = 85)	*p* value
Chest pain score, median [IQR]	1 [0–2]	2 [1–2]	**0.021**
Moderate or greater (score ≥ 3), n/N (%)	17/107 (15.9)	21/95 (24.7)	0.180

**Panel B—Radiofrequency cohort**			
Variable	General anaesthesia(N = 42)	Mild conscious sedation(N = 19)	*p* value
Chest pain score, median [IQR]	2 [1–2]	2 [2–3]	0.084
Moderate or greater (score ≥ 3), n/N (%)	8/42 (19.0)	7/19 (36.8)	0.136

## 4 Discussion

The PROMs‐PFA study is, to our knowledge, the first to prospectively and longitudinally assess patient symptoms in the early post‐AF ablation period, with a focus on the differences between PFA and thermal ablation modalities (Central Illustration 1). The key findings are:

i. Smartphone application‐based collection of early PROMs using the CARE questionnaire proved feasible, enabling structured assessment of symptom burden across multiple recovery timepoints;
ii. PFA was associated with less chest pain on Day 1 post‐ablation compared to RF and CB, remaining lower than CB at Day 7, although these differences resolved by Day 30;
iii. Awareness of a fast or abnormal heart rate was less common in the PFA group than in the RF group on Day 1;
iv. Groin discomfort was common after ablation, and more common amongst patients receiving PFA and CB ablation, consistent with a larger sheath size;
v. No significant differences were observed between ablation modalities for other symptoms, including headache, heartburn, bowel habit, or mood.

Overall, these findings suggest that while PFA may offer a modest early advantage in terms of reduced chest pain and palpitations, the broader symptom burden following AF ablation is largely similar across modalities. A major strength of PROMs‐PFA is its reflection of real‐world practice as seen in many centres worldwide [24]: all PFA and most RF procedures were performed under GA, whereas CB procedures were carried out under conscious sedation. This distribution reflects procedural requirements: GA is necessary for PFA due to skeletal muscle contractions during energy delivery, while RF benefits from GA to enhance catheter stability, highlighting that anaesthetic choice is intrinsically linked to ablation modality. Further, the cohort was unselected, and questionnaire completion rates were high, supporting the generalisability of the findings.

**Central Illustration 1:**
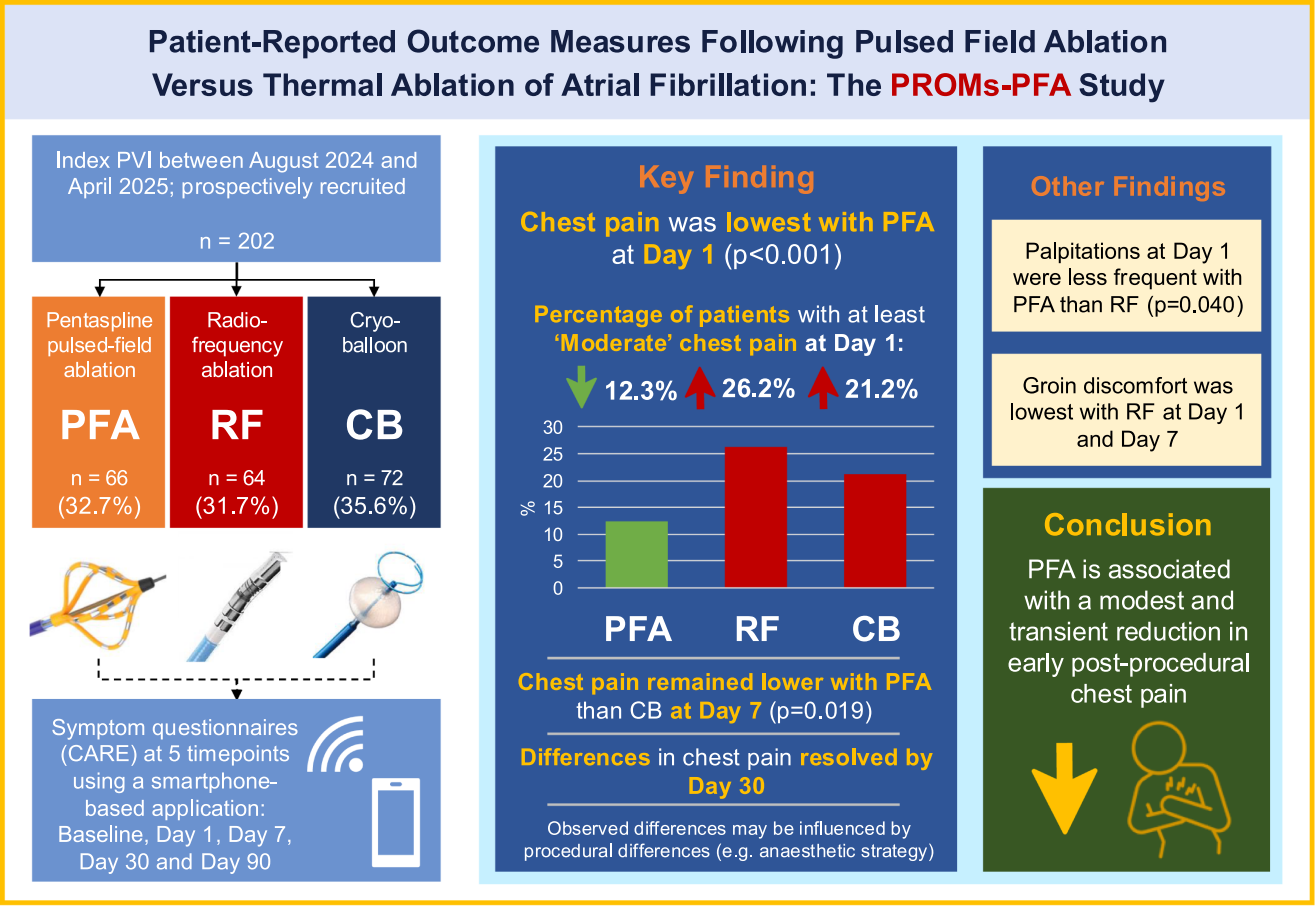
Pulsed‐field was associated with less early post‐procedural chest pain than thermal ablation.

### 4.1 PFA and Post‐Procedural Chest Pain

The lower burden of chest pain and discomfort observed with PFA at Days 1 and 7 supports the hypothesis that non‐thermal ablation may reduce early inflammatory or nociceptive symptoms. This aligns with pre‐clinical and early clinical studies reporting minimal extracardiac injury with PFA, attributed to its myocardial selectivity via irreversible electroporation [25]. The PEFCAT and IMPULSE trials demonstrated excellent acute efficacy with favourable safety profiles for PFA, noting in particular the absence of significant pericardial, oesophageal and phrenic nerve injury [26]. However, to date, post‐procedural symptomatology has not been systematically evaluated following PFA.

A small study by Wang et al. reported lower chest pain scores following PFA than RF ablation, based on a single post‐procedural survey [12]. Similarly, Isenegger et al. found a low incidence of chest pain or discomfort (7.5%) amongst 388 PFA‐treated patients [13]. Notably, neither study included repeated symptom assessments over time. Our findings therefore extend this early evidence by providing longitudinal, patient‐reported data on the trajectory and burden of post‐ablation symptoms.

In our PFA cohort, only 1 in 10 patients reported moderate (or greater) chest pain by Day 1, compared with 1 in 5 in the CB group and 1 in 4 in the RF group (Figure 2), whilst median chest pain scores were significantly lower in the PFA group overall (Table 4). Although the difference in chest pain was modest and did not persist beyond 1 week, it suggests a short‐term advantage of PFA in minimising extracardiac irritation or inflammation. Importantly, the clinical relevance of this finding is better appreciated when considering symptom severity categories: the proportion of patients reporting at least moderate chest pain was approximately two‐fold lower in the PFA group compared with thermal modalities. Importantly, these differences were modest and transient, resolving by 30 days, and did not translate into measurable differences in global quality of life at 90 days. Nevertheless, they provide insight into early recovery, which is not captured by conventional outcome measures.

The close relationship between ablation modality and anaesthetic strategy in our cohort represents an important source of potential confounding when interpreting early symptom differences. In exploratory analyses, patients treated under GA reported lower median chest pain scores at Day 1 than those treated with conscious sedation, although there was no significant difference in the proportion reporting at least moderate symptoms. Furthermore, in a stratified analysis restricted to the RF sub‐group, where both anaesthetic strategies were used, differences in chest pain according to anaesthesia were not statistically significant. These findings suggest that anaesthetic strategy may contribute to early symptom perception, but is unlikely to fully account for the observed between‐modality differences.

The higher prevalence of persistent AF and greater left atrial dilatation in the PFA cohort may have influenced symptom perception and recovery patterns, as more advanced atrial disease could alter the reporting of arrhythmia‐related symptoms. However, these factors would be less likely to explain differences in early post‐procedural chest pain, which is more plausibly related to procedural injury and pericardial or extracardiac irritation. Adjunctive PW ablation was more frequently performed in the PFA group; however, sensitivity analysis excluding these patients demonstrated consistent findings, suggesting that this procedural difference is unlikely to account for the observed differences in early chest pain.

This low chest pain burden with PFA aligns with Isenegger and colleagues' report of zero cases meeting European Society of Cardiology criteria for acute pericarditis, and only 4.4% of patients meeting single criteria such as chest pain or minor ECG changes. These findings are further supported by data from MANIFEST‐17K and EU‐PORIA registries, which reported a pericarditis incidence of ≤ 0.2% following PFA [4, 27].

Together, this growing body of evidence reinforces the concept that PFA is associated with less pericardial and pleural irritation than traditional thermal ablation. While our study did not aim to formally diagnose pericarditis, the lower chest pain burden observed amongst PFA recipients may reflect reduced subclinical pericardial inflammation, consistent with the favourable inflammatory profile described in mechanistic and registry‐based studies [4, 5, 27, 28].

### 4.2 Palpitations and Early Arrhythmic Symptoms

Palpitations are a frequent and often distressing symptom following AF ablation, commonly interpreted by patients as arrhythmia recurrence. Despite the widespread use of a ‘blanking period’ to account for transient post‐ablation arrhythmias, there is increasing recognition that patients' lives do not ‘pause’ during this time [29], and that any strategy capable of reducing early symptom burden remains clinically relevant.

In our study, palpitations were reported less frequently at Day 1 in the PFA group compared to RF. Whilst this difference was modest and did not persist beyond the first day, it may still be clinically meaningful. Similarly to the observed reduction in chest pain, this finding could reflect less local tissue irritation, which may contribute to fewer early abnormal sensations interpreted by patients as palpitations. Differences in autonomic effects between ablation modalities may also contribute, although this remains speculative and was not directly assessed in the present study. It is also plausible that the higher prevalence of persistent AF in the PFA group influenced symptom reporting. Indeed, persistent AF may be associated with reduced symptom perception, as patients become habituated to continuous arrhythmia and are less sensitive to short‐lived ectopy or atrial tachyarrhythmias. However, in the absence of systematic rhythm monitoring, it is not possible to determine whether these symptoms correspond to true atrial arrhythmia recurrence. Reported palpitations should therefore be interpreted as patient‐perceived symptoms rather than objective evidence of arrhythmia, particularly in the early post‐ablation period where symptom–rhythm correlation is known to be poor.

The transient nature of this difference observed in our cohort contrasts with findings from the SINGLE SHOT CHAMPION trial, which employed implantable loop recorders to objectively track arrhythmia recurrence [30]. PFA was associated with significantly higher AF‐free survival during a 3‐month ‘blanking period’ compared with CB (respectively, 61.9% vs. 41.9%; *p* < 0.001) [30]. Whilst our study did not include continuous rhythm monitoring, the early reduction in palpitations seen with PFA may reflect a lower arrhythmic burden in the immediate post‐procedural phase. Notably, transient arrhythmia recurrence during the blanking period may not equate to significant symptoms or patient burden, as evidenced by the limited and short‐lived differences in palpitation reports in our cohort. Previous studies have shown poor correlation between subjective palpitations and electrocardiographically confirmed arrhythmia, particularly early after ablation [31, 32]. This highlights the need to integrate continuous monitoring and PROMs in future studies to better understand the relationship between arrhythmia recurrence and patient experience.

### 4.3 Groin Discomfort and Difficulty Swallowing

Access site pain is a recognised but under‐reported contributor to post‐ablation morbidity. Groin discomfort was lowest in the RF group at Days 1 and 7, likely reflecting the smaller sheath sizes used in RF procedures. RF ablation typically requires 11 F sheaths (external diameter), compared with the larger external diameters used for pentaspline PFA (17 F) and CB (16 F). Although not typically a focus in ablation studies, differences in groin discomfort can affect mobility, sleep, and overall satisfaction during recovery. Our findings reinforce the need for optimised vascular access management, particularly in AF ablation which requires large‐bore access. Techniques such as ultrasound‐guided puncture and suture‐based haemostasis may mitigate access site morbidity and should be routinely adopted, especially in the era of expanding PFA use [33, 34, 35].

Difficulty swallowing was more commonly reported in the PFA and RF groups than in the CB cohort, with symptoms emerging early and persisting to Day 7. This finding likely reflects procedural workflow differences, particularly the greater use of TOE in the PFA and RF groups, rather than a direct effect of ablation modality itself. Prior studies have shown that TOE can induce pharyngeal or oesophageal discomfort, and in some cases mucosal trauma, particularly with prolonged probe dwell time [36, 37]. Importantly, while thermal ablation techniques carry a known risk of oesophageal injury, evidence suggests this is markedly reduced with PFA [38].

### 4.4 App‐Based PROMs Monitoring after AF Ablation

Our study demonstrates the feasibility of collecting PROMs via a smartphone application in the context of AF ablation. Engagement with the app‐based platform was high, with a 95% completion rate across the 5 questionnaires. Several factors likely contributed to this success: proactive patient engagement before the procedure, integration of push notifications and reminders within the app, and the brevity and clarity of the bespoke CARE symptom questionnaire. Importantly, this approach minimised the burden on both patients and clinical staff, offering a scalable solution for routine outcome monitoring.

The importance of PROMs in evaluating procedural efficacy and patient recovery is increasingly recognised, particularly for conditions like AF, where symptoms often guide management decisions. Traditional follow‐up methods may under‐capture transient, self‐limiting, yet distressing symptoms. While the AFEQT questionnaire is a validated and widely used tool in AF ablation trials, its design prioritises global quality‐of‐life assessment over short‐term and comprehensive symptom fluctuation. In our study, AFEQT scores at baseline and 3 months would not have detected early symptom differences between ablation modalities. In contrast, the CARE questionnaire, tailored to the post‐ablation recovery phase, identified modest yet clinically relevant differences and may offer added value in future studies assessing recovery trajectories across ablation technologies.

Overall, our findings support the integration of mobile PROMs tools into standard post‐ablation care pathways. As catheter ablation technologies evolve, particularly with the growing adoption of PFA, digital PROMs platforms may offer valuable insight into modality‐specific recovery patterns. Since rhythm monitoring is also feasible via smartphone [39], combining symptom and rhythm tracking may enable more patient‐centred follow‐up.

## 5 Strengths and Limitations

A key strength of this study is its prospective, longitudinal design, which allowed repeated, structured assessment of early post‐AF ablation symptoms across multiple timepoints. Additionally, the integration of the CARE questionnaire into a smartphone application facilitated high engagement and nearly complete data capture, providing real‐time insight into symptom trajectories that would be difficult to achieve with traditional follow‐up methods such as paper questionnaires.

Although efforts were made to standardise symptom assessment, self‐reported symptoms remain subject to bias. Post‐procedural analgesic requirements were not systematically recorded and therefore could not be analysed, which may have provided an additional objective measure of post‐procedural discomfort. Further, whilst questionnaire response rates were high, not all patients completed all questionnaires, introducing the potential for attrition bias. The high compliance rate (almost 95%) reported here may not be reflective of wider‐scale implementation, given that a clinician was monitoring responses and prompting patients via phone call where necessary. In addition, the requirement for smartphone use may have introduced selection bias, favouring a more engaged and digitally literate cohort. The absence of concurrent rhythm monitoring limits the ability to correlate symptoms (particularly palpitations) with documented arrhythmia. Consequently, reported palpitations should be interpreted as patient‐perceived symptoms rather than evidence of arrhythmia recurrence.

In addition, the analyses were not adjusted for differences in procedural characteristics between groups. This was particularly relevant for anaesthetic strategy, which was closely linked to ablation modality in our cohort, with all PFA procedures performed under GA and almost all CB procedures performed under conscious sedation, resulting in limited overlap between groups. Consequently, the observed differences in symptoms cannot be definitively attributed to the ablation modality alone. Stratified analyses to assess the potential impact of anaesthetic strategy on early chest pain were performed, but these were limited by sample size and should be interpreted with caution.

Whilst the groups were otherwise reasonably balanced, persistent AF and larger left atrial size were more common in the PFA group, which partially confounds this non‐randomised comparison and may influence symptom perception. However, these factors are unlikely to account for the observed differences in early post‐procedural chest pain, which are more plausibly related to procedural injury or pericardial irritation. In addition, our findings cannot be directly extrapolated to other PFA catheters and systems. Finally, this study was conducted in a high‐volume tertiary electrophysiology centre with established experience in AF ablation and high levels of digital engagement. The excellent PROM completion rates achieved may not be fully reproducible in other healthcare settings, particularly where staffing support or procedural workflows differ.

## 6 Clinical Implications

The findings of this study have several practical implications for clinicians involved in AF ablation. First, awareness of the early symptom profile following PFA versus thermal ablation can help inform patient counselling, setting realistic expectations regarding recovery and potential discomfort. The modest reductions in early chest pain and palpitations observed with PFA suggest that non‐thermal approaches may offer tangible short‐term advantages, which could influence patient and operator decisions when selecting ablation modality.

Although the observed symptom differences were modest and transient, reduced early chest pain and palpitations with PFA may still have practical relevance by improving patient satisfaction, facilitating earlier return to normal activities and potentially reducing unplanned healthcare contact during the recovery period. However, these outcomes were not directly assessed in the present study and require further investigation.

Second, the feasibility of smartphone‐based PROMs collection demonstrates that digital tools can be integrated into routine post‐ablation care to capture transient, self‐limiting symptoms that are often missed by traditional follow‐up. This real‐time symptom monitoring may help identify patients experiencing delayed recovery or complications and facilitate timely clinical intervention.

Importantly, the lower early symptom burden observed with PFA should not be interpreted as evidence of superior lesion durability or long‐term ablation efficacy, which were beyond the scope of the present study.

## 7 Conclusion

The prospective PROMs‐PFA study demonstrates that PFA is associated with a modestly lower early burden of post‐procedural chest pain than thermal modalities, although these differences were transient and resolved within the first month, with no differences in medium‐term quality of life. These findings highlight differences in early recovery profiles between ablation modalities and demonstrate the value of the CARE questionnaire, a PROMs tool incorporated into a smartphone‐based application and specifically designed for the early recovery phase, in evaluating the real‐world impact of evolving ablation technologies. As PFA adoption increases, symptom‐focused data should be integrated alongside traditional safety and efficacy endpoints to better inform procedural planning and optimise patient care.

## Funding

The authors have nothing to report.

## Conflicts of Interest

S.N.M.: speaking honoraria and research grants from J&J MedTech. R.A.: speaker fees from Abbott. V.L.: support from UK National Institute for Health Research scholarship award, speaker for Boston Scientific, J&J MedTech, research grants from J&J MedTech. D.G.: institutional research grants from J&J MedTech, Boston Scientific and Medtronic, and speaker fees from Boston Scientific. The other authors declare no conflicts of interest.

## Supporting information

Supporting File

## Data Availability

The data that support the findings of this study are available on request from the corresponding author. The data are not publicly available due to privacy or ethical restrictions.

## References

[1] P. Calvert , G. Y. H. Lip , and D. Gupta , “Radiofrequency Catheter Ablation of Atrial Fibrillation: A Review of Techniques,” Trends in Cardiovascular Medicine 33, no. 7 (2023): 405–415, 10.1016/j.tcm.2022.04.002. 35421538

[2] P. Calvert , W. Y. Ding , M. T. Mills , et al., “Durability of Thermal Pulmonary Vein Isolation in Persistent Atrial Fibrillation Assessed by Mandated Repeat Invasive Study,” Heart Rhythm: The Official Journal of the Heart Rhythm Society 21 (9) (2024): 1545–1554, 10.1016/j.hrthm.2024.04.061. 38636929

[3] V. Y. Reddy , E. P. Gerstenfeld , A. Natale , et al., “Pulsed Field or Conventional Thermal Ablation for Paroxysmal Atrial Fibrillation,” New England Journal of Medicine 389, no. 18 (2023): 1660–1671, 10.1056/NEJMoa2307291. 37634148

[4] B. Schmidt , S. Bordignon , K. Neven , et al., “European Real‐World Outcomes With Pulsed Field Ablation in Patients With Symptomatic Atrial Fibrillation: Lessons From the Multi‐Centre EU‐PORIA Registry,” Europace: European Pacing, Arrhythmias, and Cardiac Electrophysiology: Journal of the Working Groups on Cardiac Pacing, Arrhythmias, and Cardiac Cellular Electrophysiology of the European Society of Cardiology 25, no. 7 (2023): euad185, 10.1093/europace/euad185. 37379528 PMC10320231

[5] M. T. Mills , S. Trivedi , M. J. Lovell , et al., “Pulsed‐Field Ablation of Atrial Fibrillation With a Pentaspline Catheter Across National Health Service England Centres,” Open Heart 11, no. 2 (2024): e003094, 10.1136/openhrt-2024-003094. 39694575 PMC11667399

[6] M. Bergonti , M. T. Mills , L. Roten , et al., “Pulsed Field Ablation for Atrial Fibrillation Ablation: A European Heart Rhythm Association Survey,” Europace: European pacing, arrhythmias, and cardiac electrophysiology: journal of the working groups on cardiac pacing, arrhythmias, and cardiac cellular electrophysiology of the European Society of Cardiology 27, no. 12 (2025 December 1): euaf294, 10.1093/europace/euaf294. 41363248 PMC12686987

[7] K. A. Wood , A. H. Barnes , S. Paul , K. A. Hines , and K. P. Jackson , “Symptom Challenges After Atrial Fibrillation Ablation,” Heart & Lung 46, no. 6 (2017): 425–431, 10.1016/j.hrtlng.2017.08.007. 28923248 PMC5811184

[8] V. Jacobs , H. T. May , B. G. Crandall , et al., “Vagus Nerve Injury Symptoms After Catheter Ablation for Atrial Fibrillation,” Pacing and Clinical Electrophysiology 41, no. 4 (2018): 389–395, 10.1111/pace.13304. 29435991

[9] S. Bordignon , M. T. Mills , P. Futyma , et al., “European Heart Rhythm Association Survey on the Perceived Severity of Complications in Atrial Fibrillation Ablation: Development of a Standardized Scoring Model,” Europace: European Pacing, Arrhythmias, and Cardiac Electrophysiology: Journal of the Working Groups on Cardiac Pacing, Arrhythmias, and Cardiac Cellular Electrophysiology of the European Society of Cardiology 27, no. 11 (2025 October 31): euaf254, 10.1093/europace/euaf254. 41263149 PMC12631125

[10] M. T. Mills , G. Y. H. Lip , V. Luther , and D. Gupta , “Sex‐Based Differences in Symptomatology in the First Month Following Atrial Fibrillation Catheter Ablation,” Journal of Cardiovascular Electrophysiology 36, no. 9 (September 2025): 2271–2278, 10.1111/jce.70009. 40643051 PMC12420893

[11] R. U. Shah , J. V. Freeman , D. Shilane , P. J. Wang , A. S. Go , and M. A. Hlatky , “Procedural Complications, Rehospitalizations, and Repeat Procedures After Catheter Ablation for Atrial Fibrillation,” Journal of the American College of Cardiology 59, no. 2 (2012): 143–149, 10.1016/j.jacc.2011.08.068. 22222078 PMC5340189

[12] J. Wang , X. Wang , W. Liu , et al., “Efficacy, Safety, and Somatosensory Comparison of Pulsed‐Field Ablation and Thermal Ablation: Outcomes From a 2‐year Follow‐Up,” Journal of Interventional Cardiac Electrophysiology 69, no. 2 (2024): 201–211, 10.1007/s10840-024-01966-w. 39673645

[13] C. Isenegger , R. Arnet , F. Jordan , et al., “Incidence of Acute Pericarditis After Pulsed‐Field Ablation for the Treatment of Atrial Fibrillation,” Europace: European Pacing, Arrhythmias, and Cardiac Electrophysiology: Journal of the Working Groups on Cardiac Pacing, Arrhythmias, and Cardiac Cellular Electrophysiology of the European Society of Cardiology 26, no. 7 (2024): euae180, 10.1093/europace/euae180. 38938174 PMC11230865

[14] J. Spertus , P. Dorian , R. Bubien , et al., “Development and Validation of the Atrial Fibrillation Effect on Quality‐Of‐Life (AFEQT) Questionnaire in Patients With Atrial Fibrillation,” Circulation: Arrhythmia and Electrophysiology 4, no. 1 (2011): 15–25, 10.1161/CIRCEP.110.958033. 21160035

[15] V. Kanthasamy , R. J. Schilling , S. Horan , et al., “Development and Validation of a New Disease‐Specific Quality‐Of‐Life Measure for Atrial Fibrillation Derived From Patient Perspectives: The Atrial Fibrillation Patient‐Reported Outcome Measures (AF‐PROMs) Questionnaire,” Heart Rhythm: The Official Journal of the Heart Rhythm Society 22, no. 8 (2025): 1922–1934, 10.1016/j.hrthm.2025.02.007. 40162948

[16] G. Chu , P. Calvert , B. Sidhu , et al., “Patient Experience of Very High Power Short Duration Radiofrequency Ablation for Atrial Fibrillation Under Mild Conscious Sedation,” Journal of Interventional Cardiac Electrophysiology 66, no. 2 (2023): 445–453, 10.1007/s10840-022-01351-5. 35997848 PMC9396586

[17] P. Calvert , I. Koniari , M. T. Mills , et al., “Lesion Metrics and 12‐Month Outcomes of Very‐High Power Short Duration Radiofrequency Ablation (90W/4 s) under Mild Conscious Sedation,” Journal of Cardiovascular Electrophysiology 35, no. 6 (2024): 1165–1173, 10.1111/jce.16269. 38571287

[18] P. Calvert , M. T. Mills , P. Xydis , et al., “Cost, Efficiency, and Outcomes of Pulsed Field Ablation vs Thermal Ablation for Atrial Fibrillation: A Real‐World Study,” Heart Rhythm: The Official Journal of the Heart Rhythm Society 21, no. 9 (2024): 1537–1544, 10.1016/j.hrthm.2024.05.032. 38763378

[19] M. T. Mills , P. Calvert , L. Tidbury , et al. Figure‐of‐8 Suture With a 3‐Way Tap Versus Manual Compression After Atrial Fibrillation Ablation: The HARNESS Randomized Controlled Trial. JACC Clin Electrophysiol. 2026 Feb 12:S2405‐500X(26)00021‐6, https://www.jacc.org/doi/10.1016/j.jacep.2026.01.014. 10.1016/j.jacep.2026.01.014 41711619

[20] B. Schmidt , S. Bordignon , S. Tohoku , et al., “5S Study: Safe and Simple Single Shot Pulmonary Vein Isolation With Pulsed Field Ablation Using Sedation,” Circulation: Arrhythmia and Electrophysiology 15, no. 6 (2022): e010817, 10.1161/CIRCEP.121.010817. 35617232

[21] P. Calvert , M. T. Mills , B. Murray , et al., “Feasibility of Pulsed Field Ablation for Atrial Fibrillation under Mild Conscious Sedation,” Journal of Interventional Cardiac Electrophysiology 68, no. 7 (2024): 1429–1436, (online ahead of print), 10.1007/s10840-024-01961-1. 39623098 PMC12436502

[22] M. T. Mills , P. Calvert , C. Phenton , et al., “An Approach to Electroanatomical Mapping With a Pentaspline Pulsed Field Catheter to Guide Atrial Fibrillation Ablation,” Journal of Interventional Cardiac Electrophysiology 68, no. 4 (2025): 921–931, 10.1007/s10840-025-01980-6. 40032773 PMC12245958

[23] M. T. Mills , P. Sommer , J. Day , et al., “Characterisation of Acute Residual Pulmonary Vein Connections Using Electroanatomical Mapping During Pulsed‐Field Ablation of Atrial Fibrillation,” Heart Rhythm: the Official Journal of the Heart Rhythm Society S1547–5271, no. 25 (2025): 02616–02615, 10.1016/j.hrthm.2025.06.037. 40582685

[24] R. Garcia , V. Waldmann , P. Vanduynhoven , et al., “Worldwide Sedation Strategies for Atrial Fibrillation Ablation: Current Status and Evolution over the Last Decade,” EP Europace 23, no. 12 (2021): 2039–2045, 10.1093/europace/euab154. 34308973

[25] J. Koruth , K. Kuroki , J. Iwasawa , et al., “Preclinical Evaluation of Pulsed Field Ablation: Electrophysiological and Histological Assessment of Thoracic Vein Isolation,” Circulation: Arrhythmia and Electrophysiology 12, no. 12 (2019): e007781, 10.1161/CIRCEP.119.007781. 31826647 PMC6924932

[26] V. Y. Reddy , S. R. Dukkipati , P. Neuzil , et al., “Pulsed Field Ablation of Paroxysmal Atrial Fibrillation: 1‐Year Outcomes of Impulse, PEFCAT, and PEFCAT II,” JACC. Clinical electrophysiology 7, no. 5 (2021): 614–627, 10.1016/j.jacep.2021.02.014. 33933412

[27] E. Ekanem , P. Neuzil , T. Reichlin , et al., “Safety of Pulsed Field Ablation in More Than 17,000 Patients With Atrial Fibrillation in the MANIFEST‐17K Study,” Nature Medicine 30, no. 7 (2024): 2020–2029, 10.1038/s41591-024-03114-3. PMC11271404 38977913

[28] M. A. Popa , F. Bahlke , M. Kottmaier , et al., “Myocardial Injury and Inflammation Following Pulsed‐Field Ablation and Very High‐Power Short‐Duration Ablation for Atrial Fibrillation,” Journal of Cardiovascular Electrophysiology 35, no. 2 (2024): 317–327, 10.1111/jce.16157. 38105426

[29] M. Ruzieh , J. M. Mandrola , and A. J. Foy , “Patients' Lives Don't Pause for Blanking Periods,” American Heart Journal Plus 50 (2024): 100497, 10.1016/j.ahjo.2024.100497. 40008274 PMC11852674

[30] T. Reichlin , T. Kueffer , P. Badertscher , et al., “Pulsed Field or Cryoballoon Ablation for Paroxysmal Atrial Fibrillation,” New England Journal of Medicine 392, no. 15 (2025): 1497–1507, 10.1056/NEJMoa2502280. 40162734

[31] C. Tondo , M. Tritto , M. Landolina , et al., “Rhythm‐Symptom Correlation in Patients on Continuous Monitoring After Catheter Ablation of Atrial Fibrillation,” Journal of Cardiovascular Electrophysiology 25, no. 2 (2014): 154–160, 10.1111/jce.12292. 24102697

[32] A. Verma , J. Champagne , J. Sapp , et al., “Discerning the Incidence of Symptomatic and Asymptomatic Episodes of Atrial Fibrillation Before and After Catheter Ablation (Discern AF): A Prospective, Multicenter Study,” JAMA Internal Medicine 173, no. 2 (2013): 149–156, 10.1001/jamainternmed.2013.1561. 23266597

[33] M. T. Mills , P. Calvert , R. Snowdon , et al., “Suture‐Based Techniques Versus Manual Compression for Femoral Venous Haemostasis After Electrophysiology Procedures,” Journal of Cardiovascular Electrophysiology 35, no. 11 (2024): 2119–2127, 10.1111/jce.16417. 39233396

[34] M. T. Mills , P. Calvert , G. Y. H. Lip , V. Luther , and D. Gupta , “Outcomes of Vascular Closure Devices for Femoral Venous Hemostasis Following Catheter Ablation of Atrial Fibrillation,” Journal of Cardiovascular Electrophysiology 35, no. 8 (2024): 1656–1662, 10.1111/jce.16345. 38924288

[35] M. T. Mills , D. Gupta , V. Luther , et al., “Vascular Access Site Management During Electrophysiology Procedures: A European Heart Rhythm Association Survey,” Europace: European Pacing, Arrhythmias, and Cardiac Electrophysiology: Journal of the Working Groups on Cardiac Pacing, Arrhythmias, and Cardiac Cellular Electrophysiology of the European Society of Cardiology 27, no. 7 (2025 Jul 1): euaf117, 10.1093/europace/euaf117. 40522938 PMC12212052

[36] S. Na , C. S. Kim , J. Y. Kim , J. S. Cho , and K. J. Kim , “Rigid Laryngoscope‐Assisted Insertion of Transesophageal Echocardiography Probe Reduces Oropharyngeal Mucosal Injury in Anesthetized Patients,” Anesthesiology 110, no. 1 (2009): 38–40, 10.1097/ALN.0b013e318190b56e. 19104168

[37] R. Zeng , X. Pu , S. Chen , et al., “Oropharynx Pain, Discomfort, and Economic Impact of Transesophageal Echocardiography for Planned Radio‐Frequency Catheter Ablation in Patients With Atrial Fibrillation: A Cross‐Sectional Survey Study,” International Journal of Cardiology. Heart & Vasculature 48 (2023): 101266, 10.1016/j.ijcha.2023.101266. 37719868 PMC10500450

[38] M. Nies , J. S. Koruth , M. Mlček , et al., “Is the Esophagus Spared During Pulsed Field Ablation? Early Histopathology and In Vivo Esophageal Retraction,” Heart Rhythm: The Official Journal of the Heart Rhythm Society S1547–5271, no. 25 (2025): 02612–02618, 10.1016/j.hrthm.2025.06.033. 40581235

[39] P. Calvert , M. T. Mills , K. Howarth , et al., “Remote Rhythm Monitoring Using a Photoplethysmography Smartphone Application After Cardioversion for Atrial Fibrillation,” European Heart Journal— Digital Health 5, no. 4 (2024): 461–468, 10.1093/ehjdh/ztae028. 39081939 PMC11284012

