# Supplementary material for: Patient‐Reported Outcome Measures Following Pulsed‐Field Ablation Versus Thermal Ablation of Atrial Fibrillation: The PROMs‐PFA Study
Source: J Cardiovasc Electrophysiol. 2026 Jun 9;37(8):1685–98. doi: 10.1111/jce.70392 (PMC13475266; doi:10.1111/jce.70392)
Supplement: Supplementary file 1 [file JCE-37-1685-s001.docx]

**Supplementary Material: Contents**

**Table S1**: Cardiac Ablation Recovery Evaluation (CARE) questionnaire (Day 1 questionnaire) – pages 2-3

**Table S2**: Atrial Fibrillation Effect on QualiTy-of-life (AFEQT) scores at baseline and Day 90 in the whole cohort – page 4

**Table S3**: Symptom score over time for non-cardiac complaints by ablation modality – pages 5-6

**Figure S1**: Patient guide to downloading and using the TreatPath mobile application – pages 7-8

**Figure S2**: Chest pain score distribution from baseline to Day 90 across all ablation modalities – page 9

**Table S1**: Bespoke patient-reported outcome measure questionnaire (Day 1 questionnaire).

| Since your ablation, how troubled have you been by… | | | | | | | |
| --- | --- | --- | --- | --- | --- | --- | --- |
| ***Tick 1 box per question*** | Not at all | Hardly | A Little | Moderately | Quite a Bit | Very | Extremely |
| Chest pain or discomfort | 0 | 1 | 2 | 3 | 4 | 5 | 6 |
| An awareness of a fast or abnormal heart rate | 0 | 1 | 2 | 3 | 4 | 5 | 6 |
| Shortness of breath or difficulty breathing | 0 | 1 | 2 | 3 | 4 | 5 | 6 |
| Feeling lightheaded or dizzy | 0 | 1 | 2 | 3 | 4 | 5 | 6 |
| Headaches | 0 | 1 | 2 | 3 | 4 | 5 | 6 |
| Brain fog | 0 | 1 | 2 | 3 | 4 | 5 | 6 |
| Blurring of your vision | 0 | 1 | 2 | 3 | 4 | 5 | 6 |
| Heartburn or acid reflux | 0 | 1 | 2 | 3 | 4 | 5 | 6 |
| A sense of bloating | 0 | 1 | 2 | 3 | 4 | 5 | 6 |
| Difficulty swallowing | 0 | 1 | 2 | 3 | 4 | 5 | 6 |
| Nausea | 0 | 1 | 2 | 3 | 4 | 5 | 6 |
| Vomiting | 0 | 1 | 2 | 3 | 4 | 5 | 6 |
| Being unable to eat a full meal, or feeling very full after eating only a small amount of food | 0 | 1 | 2 | 3 | 4 | 5 | 6 |
| Constipation | 0 | 1 | 2 | 3 | 4 | 5 | 6 |
| ***Tick 1 box per question*** | Not at all | Hardly | A Little | Moderately | Quite a Bit | Very | Extremely |
| Diarrhoea | 0 | 1 | 2 | 3 | 4 | 5 | 6 |
| A change in your normal bowel movements | 0 | 1 | 2 | 3 | 4 | 5 | 6 |
| Difficulty emptying your bladder | 0 | 1 | 2 | 3 | 4 | 5 | 6 |
| Low mood | 0 | 1 | 2 | 3 | 4 | 5 | 6 |
| Anxiety | 0 | 1 | 2 | 3 | 4 | 5 | 6 |
| Groin pain | 0 | 1 | 2 | 3 | 4 | 5 | 6 |

**Table S2**: Atrial Fibrillation Effect on QualiTy-of-life (AFEQT) scores at baseline and Day 90 in the whole cohort.

| **AFEQT Score** | **Baseline**  (n=202) | **Day 90**  (n=186) | *p-value* |
| --- | --- | --- | --- |
| **Baseline AFEQT**  Symptom Score  Activity Score  Treatment Concern  Treatment Satisfaction  Overall Score | 71 [54–92]  65 [40–92]  64 [44–78]  50 [25–75]  64 [47–82] | 92 [79–100]  88 [67–98]  86 [70–97]  83 [67–100]  86 [72–95] | **<0.001**  **<0.001**  **<0.001**  **<0.001**  **<0.001** |

**Table S3**: Symptom score over time for non-cardiac complaints by ablation modality.

| **Symptom** | **PFA** | **RF** | **CB** | *p-value* |
| --- | --- | --- | --- | --- |
| **Light-headedness/dizziness**   - Baseline - Day-1 - Day-7 - Day-30 - Day-90 | 1.5 [0–2]  0.5 [0–2]  1 [0–1.25]  2 [0–2]  1 [0–1] | 1 [0–2]  0 [0–2]  1 [0–2]  2 [0–2]  0 [0–1] | 2 [0–2]  1 [0–2]  1 [0–3]  1 [0–2]  1 [0–2] | 0.908  0.676  0.271  0.667  0.761 |
| **Shortness of breath**   - Baseline - Day-1 - Day-7 - Day-30 - Day-90 | 2 [0–4]  0 [0–1]  1 [0–2]  1 [0–2]  1 [0–2] | 2 [0–3]  0 [0–2]  1 [0–2]  2 [0–3]  0 [0–2] | 1 [0–3]  1 [0–2]  1 [0–3]  1 [0–3]  1 [0–2] | 0.395  0.116  0.098  0.489  0.539 |
| **Heartburn**   - Baseline - Day-1 - Day-7 - Day-30 - Day-90 | 0 [0–1.25]  0 [0–2]  0 [0–1.25]  0 [0–1]  0 [0–2] | 1 [0–2]  0 [0–1]  0 [0–1]  0 [0–2]  0 [0–1] | 1 [0–2]  0 [0–1]  0 [0–2]  0 [0–2]  0 [0–2] | 0.712  0.522  0.658  0.160  0.277 |
| **Bloating**   - Baseline - Day-1 - Day-7 - Day-30 - Day-90 | 1 [0–2.25]  0 [0–2]  1 [0–2]  0 [0–1.25]  0 [0–2] | 1 [0–2]  0 [0–1]  0 [0–2]  0 [0–2]  0 [0–2] | 1 [0–2]  0 [0–1]  0 [0–2]  1 [0–2]  0 [0–2] | 0.573  0.703  0.633  0.487  0.994 |
| **Nausea**   - Baseline - Day-1 - Day-7 - Day-30 - Day-90 | 0 [0–1]  0 [0–1]  0 [0–0]  0 [0–1]  0 [0–0] | 0 [0–1]  0 [0–1]  0 [0–1]  0 [0–1]  0 [0–0] | 0 [0–1]  0 [0–1]  0 [0–1]  0 [0–2]  0 [0–1] | 0.554  0.936  0.323  0.389  0.610 |
| **Vomiting**   - Baseline - Day-1 - Day-7 - Day-30 - Day-90 | 0 [0–0]  0 [0–0]  0 [0–0]  0 [0–0]  0 [0–0] | 0 [0–0]  0 [0–0]  0 [0–0]  0 [0–0]  0 [0–0] | 0 [0–0]  0 [0–0]  0 [0–0]  0 [0–0]  0 [0–0] | 0.898  0.816  0.596  0.367  0.449 |
| **Change in bowel habit**   - Baseline - Day-1 - Day-7 - Day-30 - Day-90 | 0 [0–1.25]  0.5 [0–2]  0 [0–1.25]  0 [0–1]  0 [0–1] | 0 [0–1]  0 [0–2]  1 [0–2]  0 [0–2]  0 [0–1] | 1 [0–2]  0 [0–1]  1 [0–2]  0 [0–2]  1 [0–2] | 0.094  0.086  0.873  0.284  0.107 |
| **Constipation**   - Baseline - Day-1 - Day-7 - Day-30 - Day-90 | 0 [0–2]  1 [0–2]  0.5 [0–2]  0 [0–1]  0 [0–1] | 0 [0–0]  0 [0–2]  0 [0–1]  0 [0–1]  0 [0–1] | 0 [0–2]  0 [0–1]  0 [0–1]  0 [0–2]  0 [0–1] | 0.150  0.154  0.506  0.654  0.206 |
| **Diarrhoea**   - Baseline - Day-1 - Day-7 - Day-30 - Day-90 | 0 [0–1]  0 [0–0]  0 [0–0]  0 [0–0]  0 [0–1] | 0 [0–2]  0 [0–0]  0 [0–1]  0 [0–2]  0 [0–0] | 0 [0–2]  0 [0–0]  0 [0–1]  0 [0–1]  0 [0–0.5] | 0.590  0.388  0.561  0.498  0.956 |
| **Headaches**   - Baseline - Day-1 - Day-7 - Day-30 - Day-90 | 0.5 [0–2]  1 [0–2]  1 [0–3]  0.5 [0–2]  0 [0–1] | 0 [0–2]  2 [0–3]  1 [0–2]  0 [0–2]  0 [0–1] | 1 [0–2]  2 [0–2]  2 [0–3]  1 [0–3]  1 [0–2] | 0.543  0.669  0.609  0.360  0.121 |
| **Brain fog**   - Baseline - Day-1 - Day-7 - Day-30 - Day-90 | 1.5 [0–3]  1 [0–2]  1 [0–2]  1 [0–2]  0 [0–2] | 1 [0–3]  0 [0–2]  1 [0–2]  1 [0–2]  0 [0–1] | 2 [0–2]  0 [0–1]  1 [0–2]  1 [0–2]  1 [0–2] | 0.640  0.378  0.759  0.748  0.264 |
| **Blurring of vision**   - Baseline - Day-1 - Day-7 - Day-30 - Day-90 | 0 [0–2]  0 [0–2]  1 [0–1.25]  2 [0–2]  0 [0–1] | 0 [0–1]  0 [0–0]  0 [0–2]  0 [0–1]  0 [0–0] | 0 [0–2]  0 [0–0]  1 [0–2]  0 [0–1]  0 [0–1] | 0.214  0.535  0.816  0.152  0.367 |
| **Low mood**   - Baseline - Day-1 - Day-7 - Day-30 - Day-90 | 2 [0–4]  0 [0–2]  1 [0–2]  1 [0–2]  1 [0–2] | 1 [0–3]  0 [0–2]  1 [0–2]  1 [0–2]  0 [0–2] | 1 [0–2]  0 [0–1]  1 [0–2]  2 [0–2]  1 [0–2] | 0.218  0.636  0.948  0.639  0.545 |
| **Anxiety**   - Baseline - Day-1 - Day-7 - Day-30 - Day-90 | 1 [0–3]  0 [0–2]  1 [0–2]  1 [0–2]  0 [0–2] | 2 [0–3]  1 [0–2]  1 [0–2]  1 [0–3]  1 [0–2] | 2 [0–3]  0 [0–1]  1 [0–2]  1 [1–2]  1 [0–2] | 0.499  0.069  0.718  0.170  0.612 |

*CB: cryoballoon; PFA, pulsed-field ablation; RF, radiofrequency. p-values are corrected for multiple hypothesis testing via the Bonferroni method*

**Figure S1**: Patient guide to downloading and using the TreatPath mobile application.

**Figure S2**: Chest pain score distribution from baseline to Day 90 across all ablation modalities.
